# Energy and economic benefits from economies of scale in intercity freight transportation

**DOI:** 10.1038/s44333-025-00028-6

**Published:** 2025-03-28

**Authors:** Philip Krammer, Andreas W. Schäfer

**Affiliations:** UCL Energy Institute, Bartlett School of Environment, Energy and Resources, University College London, London, UK

**Keywords:** Energy economics, Energy efficiency, Energy and society

## Abstract

Since the beginning of motorization, intercity freight transportation modes have carried an ever-increasing load and experienced a nearly continuous decline in average unit costs and energy intensity. Using a unique dataset, we demonstrate the tight, inverse relationships between the average load carried with average transport unit costs and energy intensity, which are invariant across mode, space, and time, for the countries and time periods considered. Our subsequent statistical analysis concludes that—over the last 30–35 years—economies of scale have contributed to 65–85% of the decline in energy intensity, depending on the transport mode, with only the remaining share being due to technological progress. Significant further reductions in average unit costs and energy intensity due to increasing economies of scale seem possible for particularly surface transport modes. Instead of technological advances, their realization could require legal and infrastructure adjustments.

## Introduction

Between 1920 and 2019, the average cost per tonne-km of US freight railways declined by 77%, while the amount of energy consumed per tonne-km dropped by 95%. During the same 100-year period, the average load carried increased more than fivefold^1^. Significant reductions were observed also for the US freighter aircraft fleet. Between 1991 and 2019, average unit costs declined by 34% and energy intensity by 38%, while the average load increased by 60%^2^. Large reductions in both average unit costs and energy intensity can be expected to have occurred also for US intercity freight trucks, the average load of which increased by a factor 10 between 1925 and 1995^3,4^.

These unit cost and energy intensity reductions were enabled by mainly two factors, technological progress and an increase in average vehicle load. Technological progress can be incremental or disruptive; over the past, it was a combination of both^5^. Examples of disruptive technological change include the transition from steam to diesel locomotives, the shift from aircraft piston to jet engines, and the switch from gasoline to diesel engine trucks. In contrast, incremental technological progress can be exemplified by improving the energy recovery of diesel-electric locomotives, increasing the bypass ratio of turbofan engines, or recovering an increasing share of thermal energy in the exhaust gas of diesel engines. Both disruptive and incremental technological progress led also to advanced braking systems (e.g., ref. ^6^). These and other improvements contributed to the second factor leading to reduced freight transport energy intensity, that is, larger freight transport systems that further exploit economies of scale.

Economies of Scale (EoS) occur when the cost of production increases to a lesser extent than economic output, that is, the costs per unit output decline with increasing scale. For example, a freight train operator adding an extra carload will experience higher variable costs (maintenance and fuel), whereas the fixed costs (labor and capital) remain unchanged. However, the decline in fixed costs per tonne-km is stronger than the increase in variable costs per additional tonne-km, thus leading to a decline in average unit costs (until the emergence of diseconomies of scale). These economies of vehicle size are often reinforced by economies of distance, as longer trains, heavier trucks, and larger aircraft operate typically over longer distances, thus reducing the fixed costs per tonne-km, such as those associated with train shunting or terminal facilities use^7^. The higher the share of fixed costs to total costs, the stronger the average unit cost reductions and thus the larger the EoS.

Most research in freight transport unit costs with respect to scale economies has focused on individual transport modes, increasingly container ships (e.g., refs. ^8,9^), rather than modal comparisons. In contrast, much of the research in freight transport energy intensity relates to modal comparisons, reporting either averages (e.g., refs. ^10,11^) or ranges (e.g., ref. ^12^) at aggregate levels. Whereas these energy intensity studies are useful benchmarks, their aggregate nature ignores important effects at higher levels of resolution. An example of a more disaggregate study is the 1982 CBO report, which distinguished energy intensities by commodity groups and other factors^13^. Ref. ^14^ builds upon that study, providing updated intensities, using a similar segmentation. The CBO study was the basis also for an analysis, which introduced the average vehicle load as a quantifiable explanatory variable to describe commodity groups and their impact on freight transport energy intensity (ref. ^15^). Here we refine this analysis and extend it to unit costs.

Table 1 describes the dataset underlying this study. The intercity freight transportation data builds upon 10 countries and two aggregated country groups of the European Union (trucks) and global total (ocean vessels), altogether encompassing a period from 1925 through 2019 and making use of around 40 data sources (see Supplementary Table 1 for a detailed description of the dataset).

**Table 1:** Summary statistics of the data underlying this study between 1990 and 2019

Mode	Period	No. of observations	Unit costs $(2021)/tonne-km		Energy intensity^a^ MJ/tonne-km		Average load^b^ tonnes	
			Mean	10th–90th IPR	Mean	10th–90th IPR	Mean	10th–90th IPR
Trucks^c^	1999–2019	372	0.114	0.058–0.170	2.19	1.05–3.20	10.9	3.12–20.2
Railways	1990–2019	205	0.026	0.017–0.033	0.241	0.191–0.286	979	795–1,180
Ocean Vessels	2010–2012	2406	0.004	0.002–0.007	0.136	0.061–0.208	26.6 × 10^3^	7.49–43.6 × 10^3^
Freighter Aircraft	1991–2019	774	0.844	0.228–1.74	16.4	8.00–31.1	33.1	10.2–65.3
Pipelines	2003–2019	112	0.021	0.008–0.053	0.408	0.200–0.683	1.15 × 10^6^	0.457–1.83 × 10^6^

For simplicity, we aggregate all container, bulk, and oil carriers into one ocean vessel category and oil and gas pipelines into one class of pipelines. Moreover, for better comparability, we include data only from 1990 to 2019.

^a^No data available for gas pipelines; data relates to oil pipelines.

^b^For railways, average load per locomotive; for pipelines, average load per pumping/compressor station.

^c^188, 184, and 372 observations for unit costs, energy intensity and the combined average load dataset.

The summary statistics in Table 1 indicate substantial differences in the mean values of unit costs and energy intensity by mode of transport. Aircraft account for the highest average unit cost of $0.844/tonne-km, whereas ocean vessels offer the lowest value of $0.004/tonne-km, a ratio of about 200. (Container vessels would increase unit costs only marginally to $0.005/tonne-km). The differences in average energy intensity are of similar magnitude, that is 16.4 MJ/tonne-km for aircraft vs. 0.14 MJ/tonne-km for ocean vessels. At the same time, ocean vessels carry more than 800 times as much cargo weight than aircraft on average. The variations in unit costs and energy intensity for a given mode are still significant but smaller. The 90th percentile figures are typically three to six times larger than the 10th percentile.

Importantly, the unit cost and energy intensity statistics in Table 1 depend on the data included in the sample and are context-specific. As we will show below, they are meaningless on their own without accounting for their dependence on average load.

## Results

### Unit costs

**Fig. 1:**
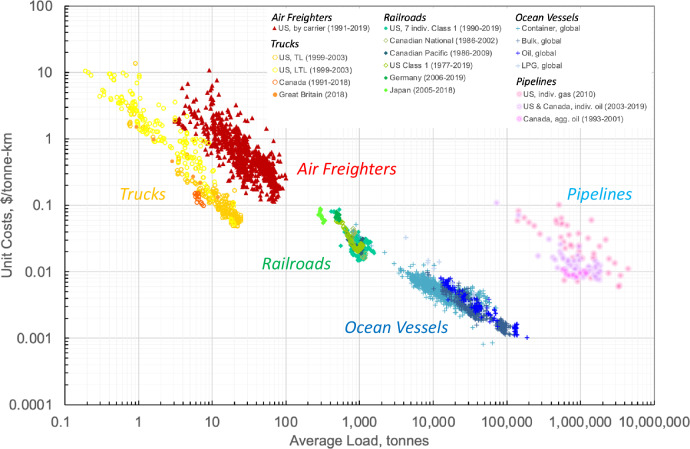
Average transportation unit costs versus average vehicle load for trucks, railways, waterways, aircraft, and pipelines. See Supplementary Information for a description of the data sources. The average load is defined per traction unit (pumping or compressor station for pipelines). Jointly, the five modes cover a four-order-of-magnitude difference in unit costs and a seven-order-of-magnitude difference in average vehicle load. On one extreme are small trucks transporting less than one tonne of cargo at a cost of around $10 per tonne-km. On the other extreme are marine tankers, transporting oil in excess of 100,000 tonnes of cargo at a cost of about 0.1 Cents per tonne-km. The prospects of benefitting from these scale economies have been the decisive force for increasing vehicle size. See Supplementary Table 2 for the composition of unit costs. US truck data relates to individual trucking companies; some of which may use light trucks.

The decline in transport unit costs with increasing average load applies to all freight modes and is invariant across space and time as shown in Fig. 1. Each of the five freight transport modes is represented by a distinct trajectory. As can be seen, the variation in unit costs for each mode, already discussed in the context of Table 1, is significant and can be attributed mainly to different levels of average load. If taking into account this relationship, the value of unit costs becomes largely independent of the number of observations in contrast to the mean values shown in Table 1.

In Fig. 1, pipelines represent the highest cost trajectory, which can be attributed to their comparatively high amount of fixed costs per tonne-km. As the latter are lowest for trucks, they define the lowest cost trajectory; in between are those of aircraft, railways, and waterways. Altogether, at a given vehicle load, unit cost differences range over three orders of magnitude. Such inherent cost differences can be overcome only through a massive increase in scale: pipelines require an average load of well above 100,000 tonnes and more than 1,000,000 tonnes per pumping or compressor station to compete with railroads and ocean vessels, respectively, everything else equal. Hence, not surprisingly, each transportation mode operates only within a specific range of scales, within which it is economically competitive, provides a unique service, and/or is constrained by technological or infrastructure limitations.

The share of fixed costs to total costs along with the costs of producing an additional tonne-km then determine the rate of decline in average transport unit costs with increasing scale. These cost components’ combined effect is highest for railroads and pipelines, but lowest for container ships, thus resulting in the highest and lowest slope, respectively. According to our econometric analysis, a 10% increase in average trainload leads to an 8.7% ( = 1–1.1^−0.95^) unit cost reduction (see “Methods”). On the other extreme, container ships, the mode with one of the lowest shares of fixed costs, experience the lowest scale elasticity of −0.57, translating into a 5.3% decline in transport unit costs in response to a 10% increase in average shipload, as shown in the left data column in Table 2 (unfortunately, robust pipeline data is difficult to access and thus our estimates of related scale elasticities are not sufficiently reliable and not reported).

**Table 2:** Scale elasticity of unit costs and energy intensity for four intercity transportation modes

	Scale elasticity of unit costs	Scale elasticity of energy intensity
Road	−0.63 to −0.88^a^	−0.49 to −0.61^b^
Rail	−0.95^c^	−0.77^c^
Sea	−0.57 to −0.75^d^	−0.40 to −0.58^e^
Air	−0.91^f^ (−1.07)^g^	−0.59^f^ (−0.77)^g^

See “Methods” for underlying regression analysis.

Due to data limitations, the underlying data is composed of multiple regions over different time frames (see Supplementary Information).

^a^Left number: Canadian intercity transport between 2004 and 2018; right number: US intercity truckload transport between 1999 and 2003.

^b^Left number: multiple countries between 1989 and 2018 (see Supplementary Table 1); right number: Australian rigid and articulated trucks between 1985 and 2019.

^c^Seven Class I railroads in the US between 1990 and 2019.

^d^Left number: container ships between 2010 and 2012; right number: bulk and oil carriers between 2010 and 2012.

^e^Left number: container ships between 2010 and 2012; right number: oil carriers between 2010 and 2012.

^f^Dedicated air freighters in US domestic and international transport between 1991 and 2019 without fixed effects

^g^Dedicated air freighters in US domestic and international transport between 1991 and 2019 with fixed effects, accounting for aircraft types. In this case, the estimated elasticity corresponds to that of the payload. See Supplementary Tables 3 and 4 for detailed statistical results.

### Energy intensity

Energy intensity, i.e., energy use per revenue tonne-km (E/RTK), can be broken down into energy use per vehicle-km traveled (E/VKT) and the inverse vehicle load (VKT/RTK), as per identity 1.

1 $${\rm{E}}/{\rm{RTK}}={\rm{E}}/{\rm{VKT}}\,{{\cdot }}\,{\rm{VKT}}/{\rm{RTK}}$$

An increase in vehicle load (RTK/VKT) will result in a lower energy intensity, despite the simultaneous increase in E/VKT due to the heavier vehicle. This can be more easily seen when breaking down vehicle load into load factor (RTK per available RTK) and vehicle capacity (available RTK per VKT), as shown in identity 2.

2 $${\rm{RKT}}/{\rm{VKT}}={{\rm{RTK}}/{\rm{RTK}}}_{{\rm{avail}}}\,{\cdot }\,{{\rm{RTK}}}_{{\rm{avail}}}/{\rm{VKT}}$$

A rise in the load factor is always larger than the associated increase in vehicle energy use, in part because cargo accounts for only a fraction of total vehicle weight, thus leading to a decline in E/RTK. A rise in vehicle capacity will cause also energy intensity to decline, as an increase in engine or vehicle size implies that its volume grows more strongly than its surface area, that is, cube vs square. Because engine power scales with volume and losses (friction and heat transfer) scale with surface area, the ratio of engine power to losses and thus thermal efficiency increases with engine size, everything else equal^15,16^. Similarly, the volumetric carrying capacity of larger surface vehicles grows more strongly than vehicle resistance (aerodynamic drag, water resistance, etc.), which scales with their surface area, that is cube versus square. Hence, vehicle resistance per volume of cargo declines also with vehicle size (for aircraft, the volumetric carrying capacity-related benefit is likely to be smaller, as the declining skin friction drag per unit cargo volume is at least partly offset by the increasing induced drag of the larger and thus heavier aircraft). Thus, as a consequence of partly the economies of vehicle size, an increase in vehicle load carried will translate into lower energy intensity and reduced fuel-related unit costs, hence contributing to the average unit cost reduction shown in Fig. 1.

**Fig. 2:**
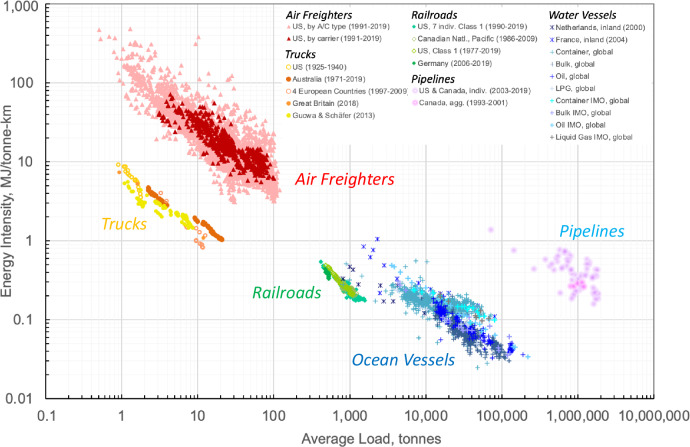
Average transportation energy intensity versus average vehicle load for trucks, railroads, water vessels, freighter aircraft, and crude oil pipelines. See Supplementary Information for a description of the data sources. Average vehicle load is expressed by the ratio of revenue tonne-km per traction system-km. For oil pipelines, it corresponds to the annual throughput in tonnes divided by the number of pumping stations. Large container ships are designed for and operated at higher speeds than bulk carriers and oil tankers, resulting in a higher energy intensity. The comparatively low slope of container ships can be attributed to their elevated speeds and associated increase in energy intensity over the 2010–2012 period. Some data points relate to fleet averages and may include light trucks.

As with average unit costs, Fig. 2 shows distinct trajectories of energy intensity over average load for each of the five transportation modes. The same observations apply with respect to the dependence of modal energy intensity on average load and the relative independence of the number of observations if this relationship is taken into account. Similar to Fig. 1, the decline in energy intensity with increasing scale is invariant across space and time. Furthermore, the positions of the modal trajectories relative to each other are analogous to those in Fig. 1: at a given vehicle load, pipelines represent the most energy-intensive transport mode, whereas trucks experience the lowest energy intensity. The comparatively low trajectory of trucks implies that the largest trucks can reach an energy intensity level that is comparable to that of small freight trains, despite the order-of-magnitude smaller vehicle load. As already observed for unit costs, each mode operates only within a window of opportunity, which is defined by its economic competitiveness, the provision of a unique service, and/or limitation by technology or infrastructure.

The energy intensity of air freighters shown in Fig. 2 is higher compared to that of belly freight in passenger aircraft (not shown here). This is a consequence of the different nature of these intensities. The belly freight-related energy intensity is marginal in nature, whereas that associated with air freighters represents an average intensity. Hence, if loading extra freight onto an air freighter with a load factor of, say, 70%, the energy intensity associated with that extra load would be also smaller than the average energy intensity—a consequence of scale economies. Similar considerations apply to unit costs.

As with the development of unit costs in Fig. 1, the mode-specific trajectories in Fig. 2 experience different rates of decline. In the case of unit cost reductions, the underlying factors relate to mainly economics; here they relate to mainly physics as represented by the factor E/VKT in identity 1. The larger the increase in this factor as a result of an increase in vehicle load, the lower the rate of decline of the modal trajectories in Fig. 2. Conversely, if E/VKT remained essentially unchanged in response to an increase in vehicle load, the energy intensity benefits of the latter would be fully exploited. As can be seen, railroads get closest to this condition, experiencing the steepest decline in energy intensity with an increasing load. Because of the significantly lower rolling resistance coefficient of railroad steel wheels on steel rails versus truck pneumatic tires on roads, a given increase in vehicle load will lead to a smaller increase in the rolling resistance of railroads compared to trucks, everything else equal. As a result, the change in E/VKT is smaller and the reduction of E/RTK stronger for railroads. This effect will only slightly be moderated by the different share of rolling resistance to total resistance for two modes. Similar comparisons can be carried out for all other modes.

Our statistical analysis suggests the scale elasticity of energy intensity to be between −0.77 (railroads) and −0.40 (container ships) as shown in the right data column in Table 2. In agreement with the modal trajectories in Figs. 1 and 2, the ranking of the scale elasticity of unit cost and energy intensity in Table 2 is identical for both modes. A comparison of the scale elasticities of unit costs and energy intensity also shows that the former are larger than the latter. This was to be expected, as those of unit costs include the energy intensity effect plus that of other operating cost categories, such as labor and capital costs.

### The contribution of economies of scale to historical energy intensity reductions

**Fig. 3:**
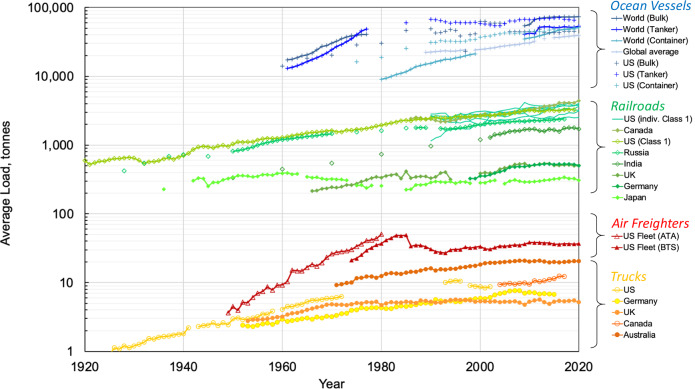
Increase in average vehicle load over time for trucks, freighter aircraft, railways, and ocean vessels. For railways, the average load per freight train is shown. For ocean vessels, the average load is expressed as the average deadweight tonne. ATA and BTS references for US freighter aircraft fleet relate to data source. See Supplementary Information for a description of the data sources.

The systematic decline in transport unit costs with increasing vehicle load has led to the use of ever larger vehicles in intercity freight traffic. Figure 3 shows the continuous increase in average load carried, ranging from a factor of 5–10 for US railroads and freighter aircraft over the past 70–100 years. Data from other countries are aligned with the US trends, although the available time series are typically shorter.

The historical increase in average load carried in Fig. 3 in combination with the scale elasticities in Table 2 allows estimating the decline in freight transport energy intensity due to increasing economies of scale. Because disruptive technological innovations, such as the transition from steam to diesel-electric locomotives, the shift from piston to jet engine aircraft, and the displacement of truck spark-ignition engines by diesel engines, caused shifts in the respective modal energy intensity trajectory in Fig. 2, we estimate the decline in energy intensity due to economies of scale over only a 30–35-year time period, after which these changes have materialized.

Our analysis, summarized in Table 3, suggests that over the last three decades, economies of scale contributed most to the reduction in energy intensity. Among the three modes, the contribution of economies of scale was highest for railroads, culminating at 85% for energy intensity reductions; only the remaining 15% can be attributed to technological change. A similar analysis for unit costs was not possible due to data limitations.

**Table 3:** Percentage total reduction in energy intensity over study period, and the approximate percentage contributions due to economies of scale in intercity freight transportation

	% Change in energy intensity	% Contribution of EoS
Road (1985–2019)	-27	80
Rail (1990–2019)	-37	85
Air (1991–2019)	-38	65

EoS contribution based on elasticities in Table 2. Road data relate to Australian rigid and articulated trucks, whereas rail and aircraft represent the US market. No time series data was available for water vessels and pipelines, which had to be excluded. See Supplementary Table 5 for detailed results.

### Limits to growth in average load carried

Given the significant historical reductions in energy intensity due to economies of scale, the question arises to what extent this trend can continue to be exploited in future. In other words, to what extent can vehicle size continue to grow before diseconomies of scale begin to dominate. We discuss the occurrence of potential limitations for railways, trucks, aircraft, and water vessels.

#### Railways

The global railway system can be broken down into essentially two groups. The first group consists of large countries with vast natural resource endowments, such as the US, Canada, Russia, and India and is characterized by very high trainloads of 1800 tonnes (India) to 4100 tonnes (Canada), which are enabled by largely dedicated railway tracks^17,18^. Such high trainloads then translate into long trains, with the average length of US freight trains being around 2 km, and the most productive trains reaching nearly 5 km^19^. In contrast, the second group consists of smaller and less mineral-rich countries, where railway tracks are being shared with faster and more frequent passenger trains. Consequently, the maximum train length and thus load is limited by infrastructure constraints, such as the lack of long sidings. Average trainloads in this second group of countries are only 10–15% compared to those in the US, ranging from 309 in Japan and 467 in South Korea to 500 tonnes in Germany and Great Britain^20–24^.

In the first group of countries with dedicated railway tracks, for the extreme case of very long freight trains, the diseconomies of scale may have already emerged as a consequence mainly of structural change in the economy. In the US, the changing cargo composition from minerals to higher-value shipments (ref. ^1^) has increased the importance of shorter and more reliable delivery times, which favors shorter trains, as “long trains take longer to assemble and disassemble in freight yards and can lead to delays on main lines”^19^. Indeed, between 2010 and 2019, the increase in average trainload has begun to slow down markedly, translating into roughly constant levels of energy intensity^1^. In addition, very long freight trains have raised safety concerns^25^. Meanwhile, train lengths are promoted to grow in the second group of countries. For example, within the EU, member countries plan to increase the train length to the general maximum of 740 m and to 835 m on specific routes^26,27^. Similar plans exist in other countries, including the UK and South Korea^28,29^. Realizing these plans would lead to an increase in trainload by 25% in Germany to 52% in South Korea^29,30^. In light of the scale elasticities in Table 2, this increase would result in a 19–33% reduction in unit costs and into a 16–28% reduction in energy intensity, respectively.

#### Trucks

Truck weight in the US was first regulated in 1913 on a state level due to concerns over road damage. The Federal laws from 1974, which limit the weight to 36.3 tonnes (80,000 lbs) for five-axle combination trucks, are still in force today, partly because of lobbying from the railroad industry against any further increase to limit competition^31^. However, state exemptions allow significantly higher limits, the highest being in Michigan with 74.5 tonnes for 11 axle-trucks^32^. A similar range of truck weight limits exists in European countries, with the highest allowed gross vehicle weight (GVW) of 76 tonnes in Finland^33^. Still, higher weight limits exist in Brazil with 91 tonnes^34^ and Australia, where 18-axle road trains experience a maximum weight limit of 135.5 tonnes^35^. In all countries, gross vehicle weight limits are complemented by those related to the maximum axle load.

The key diseconomies of scale associated with larger trucks are related to infrastructure damages; pavement damages roughly scale with the fourth power of a vehicle’s axle load (the GVW divided by the number of axles), whereas bridge damage depends mainly on the GVW, the number of axles, and axle distance^36^. Although a shift to even larger trucks would further increase these diseconomies of scale (requiring the strengthening of bridges), the multiple national initiatives exploring the benefits and costs of a further increase in truck weight and associated trials (ref. ^34^), culminating with those in Finland and Sweden with vehicles reaching a maximum weight of 104 tonnes (ref. ^37^), seem to suggest that these diseconomies of scale are considered to be smaller than the benefits generated by using larger vehicles, that is, lower unit transport costs, reduced levels of traffic congestion, as well as lower energy and CO_2_ intensity.

Nor changes to industrial production models seem to halt the trend towards increasing vehicle size and load. In response to Just-in-Time (JiT) inventory management systems, which rely on more frequent, smaller, and JiT-deliveries to reduce inventory costs, supply chains have been reorganized and now rely on consolidation runs across multiple suppliers instead of carrying out economically wasteful individual shipments^38^.

#### Aircraft

Because of the limited market size and utilization, all civilian freighter aircraft have been derived from their passenger counterparts, either via freighter programs or conversions from retired passenger vehicles. Thus, from today’s perspective, the future average freighter aircraft size will likely continue to depend on the type of passenger aircraft becoming available for freighter programs or conversions. The world’s largest passenger aircraft, the Airbus A380 and Boeing B747 halted production in 2021 and 2022, respectively. The next larger air freighter is the Boeing B777F with a payload capacity of 102.8 tonnes. Applying the average US load factor of 50% leads to the fleet-average 2019 load of around 51 tonnes, thus suggesting that the maximum aircraft load has been reached. In their market outlooks for narrow- and wide-body aircraft, both Airbus and Boeing anticipate the 2020 global fleet composition also to remain essentially unchanged by 2040^39,40^. Everything else equal, this projection would imply currently observed average loads to prevail into the future. Hence, the opportunities for further EoS-induced reductions in aviation energy intensity seem very limited.

#### Water vessels

The increase in average carrying capacity of the global shipping fleet was driven mainly by larger container ships, originating from a strong growth in containerized international trade. The largest container vessel as of 2023, the MSC Irina, has a DWT of 240,000, nearly five times that of the average fleet^41^. Such very large ships already experience diseconomies of scale, such as longer loading and unloading times, which translates into higher port fees. Very large ships can also lead to higher logistics costs and are more prone to accidents and loss of cargo. In addition, ship size is technically limited by the restrictions of waterways, foremost the Suez Canal, unless more expensive longer routes or multimodal transport are chosen.

However, even if assuming the size of the largest ships not to continue to increase any further, smaller ships in the existing fleet are likely to be replaced by larger counterparts, especially in the container ship segment, thus leading to a continuous increase in average load, everything else equal, and a corresponding decline in energy intensity.

## Discussion

The quest for higher profits in intercity freight transportation has been a force of change, driving both technological progress and exploitation of economies of scale. Over the past 30–35 years, and across our dataset, both factors have caused unit costs and energy intensity to decline by 1.5–2.1% per year and by 0.9–1.7% per year, depending on the mode. According to our analysis, and across all modes examined, most of the energy intensity reductions during that period can be attributed to increasing economies of scale. Its significance is most extreme in the case of railroads, where economies of scale accounted for 85% of the decline in energy intensity since 1990; only the remaining 15% can be attributed to technological progress. The observed declining trend in freight transportation energy intensity is in stark contrast to passenger mobility, where societal trends have systematically led towards higher levels of energy intensity^42^.

Using our dataset, we identified distinct modal trajectories of unit costs and energy intensity with vehicle load. Because of their dependence on vehicle load, a simplistic comparison of average unit costs and energy intensity across modes, as often done in the literature, is meaningless—as a minimum, vehicle load would need to be considered, too. (But even a comparison of unit costs and energy intensity of entire modal trajectories can be misleading, as these can provide a different type of service, e.g., high-speed air freight vs. slow-speed rail shipments). Each modal trajectory has been limited to only a specific range of scales, within which the respective mode is economically competitive, provides a unique service, and/or is constrained by technological or infrastructure limitations. Over the past 100 years, the latter two constraints have been relaxed through technological progress and infrastructure investments, thus expanding the range of scales by up to one order of magnitude on average.

From today’s perspective, an increase in average load by a similar extent is unlikely to materialize in future. However, our analysis identifies scope for further considerable exploitation of economies of scale, thus yielding continuous reductions in unit cost and energy intensity. The amount then depends on mode and country. For example, realizing the anticipated increase in freight trainloads by 25% in Germany and 52% in South Korea would lead to a 16% and 28% reduction in energy intensity, respectively (based upon the estimated US scale elasticity of −0.77). Doubling the maximum truckload from the current US Federal limit from 36.3 tonnes to that of Michigan state would result in a 32% reduction in energy use per tonne-km for the largest truck size segment (based upon the mean value of −0.55 of the estimated range in scale elasticities). Energy intensity reductions of this size are at the technological frontier and above of what could be achieved through efficiency improvements based upon today’s state-of-the-art truck and railway technology, respectively (e.g., ref. ^43^). In addition, whereas energy efficiency improvements through technological progress are likely to lead to ever-rising unit costs as an increasing amount of the remaining potential is being exploited, they are even negative if realized by economies of scale. However, exploiting these opportunities could require investments for modifying the rail infrastructure and for strengthening road bridges. At the same time, legislation would need to be changed to relax current limits. In contrast, our analysis suggests that the fuel burn reduction potential of freighter aircraft due to increasing scale economies is limited; fuel efficiency improvements due to technological progress offer a significantly larger opportunity^44^.

Diseconomies of scale could have become apparent for the longest freight trains in North America and the largest water vessels, manifested partly by the comparatively low speed of handling higher-value goods and in terms of traffic safety. However, these very long and large vehicles represent only a small percentage of the respective global fleet and a significant potential exists for increasing the scale of the many other, significantly smaller counterparts.

Although significant, the identified economies of scale-related reductions are far from leading the sector towards the net-zero CO_2_ emissions goal by mid-century, implicitly required by the Paris Agreement. Yet, economies of scale are an important enabler, as a lesser amount of investments needs to be stimulated for producing net-zero-carbon fuels. Economies of scale-induced energy intensity reductions are particularly attractive, as these will happen simply as a result of market forces.

This study had to build upon a sparse amount of data. A more comprehensive dataset would enable estimating the contribution of economies of scale to the historical decline in also unit costs. Furthermore, an estimate of the contribution of economies of scale to reductions in energy intensity of railways and freighter aircraft was possible only for US conditions, and that of trucks only for the Australian setting. A more complete dataset would allow quantifying cross-country differences due to those in fleet characteristics, levels of traffic congestion, and other factors. However, these data limitations don’t prevent us from concluding the existence of tight, inverse relationships between the average load carried with average transport unit costs and energy intensity, for those countries and time periods considered, and the dominant role of scale economies in reducing freight transport energy intensity over the past three decades.

## Methods

To quantify the economies of scale via elasticity β_1,_ the unit costs are specified as

3 $${\mathrm{ln}}\frac{C}{{RTK}}={\beta}_{0}+{\beta }_{1}\,{\mathrm{ln}}\frac{{RTK}}{{VKT}}+\,{\beta }_{2}{\mathrm{ln}}\,{P}_{{Fuel}}+\sum _{i}{\beta }_{i}{\mathrm{ln}}\,{V}_{i}+\varepsilon$$

and energy economies of scale as

4 $${\mathrm{ln}}\frac{E}{{RTK}}={\beta }_{0}+{\beta }_{1}{\mathrm{ln}}\frac{{RTK}}{{VKT}}+\,\sum _{i}{\beta }_{i}{\mathrm{ln}}{V}_{i}+\varepsilon$$

where C represents costs, RTK revenue tonne-km, P_Fuel_ fuel price, E energy use, V other variables (number of locomotives per train, year, indicator variables, for example, with respect to aircraft type), and ε the error term.

Because of the aggregate nature of RTK/VKT, β_1_ accounts for economies of vehicle size and distance^7^. As a sensitivity case, we estimate economies of scale also with respect to the size of aircraft and railways (for which these data are available), by decomposing RTK/VKT into the product of the vehicle load factor (RTK/RTK_available_) and vehicle capacity (RTK_available_/VKT), with VKT representing vehicle-km traveled, including empty trips. This is possible only for aircraft and railroads operating in the North American market due to the aggregate nature of the other data.

## Supplementary information

Supplementary Information

## Data Availability

The data that support the findings of this study are available from the corresponding author, A.W.S., upon reasonable request.

## References

[1] Association of American Railroads (AAR). In *Railroad Facts Washington, DC* (Association of American Railroads, 1934–2021).

[2] Bureau of Transportation Statistics (BTS). In *Air Carrier Summary Data, Form* 41 (Bureau of Transportation Statistics, Department of Transportation, 1991–2019).

[3] Barger H. *The Transportation Industries, 1889-1946: A Study of Output, Employment, and Productivity* (NBER Books, 1951).

[4] Davis S. C. *Transportation Energy Data Book: Editions 12-20* (Office of Transportation Technologies, US Department of Energy, 1992–2000).

[5] Koh, H. & Magee, C. L. A functional approach for studying technological progress: extension to energy technology. *Technol. Forecast. Soc. Change* **75**, 735–758 (2008).

[6] Federal Railroad Administration (FRA). *ECP Brake System for Freight Service, Final Report* (US Department of Transportation, 2006).

[7] Pienaar, W. J. Opportunities for the achievement of economies of scale in freight transport. *Corp. Ownersh. Control* **11**, 161–174 (2013).

[8] Cullinane and Khanna. Economies of scale in large container ships. *J. Transp. Econ. Policy* **33**, 185–207 (1999).

[9] International Transport Forum. *The Impact if Mega-Ships* (OECD, Paris, 2015).

[10] Tolliver, D., Lu, P. & Benson, D. Comparing rail fuel efficiency with truck and waterway. *Transp. Res. Part D.* **24**, 69–75 (2013).

[11] Texas A&M Transportation Institute. *A Modal Comparison of Domestic Freight Transportation Effects on the General Public: 2001–2019* (Maritime Transportation Research and Education Center (MarTREC), 2022).

[12] ICF International. *Comparative Evaluation of Rail and Truck Fuel Efficiency on Competitive Corridors*, (Federal Railroad Administration, 2009).

[13] Congressional Budget Office. *Energy Use in Freight Transportation* (Staff Working Paper, 1982).

[14] Vanek, F. Mode and commodity perspectives on U.S. freight energy consumption and CO_2_ emissions: Insights and directions for improvement. *Int. J. Sustain. Transp.* **13**, 741–760 (2019).

[15] Gucwa, M. & Schäfer, A. The impact of scale on energy intensity in freight transportation. *Transp. Res. Part D: Transp. Environ.* **23**, 41–49 (2013).

[16] Schäfer, A. W. & Yeh, S. A holistic analysis of passenger travel energy and greenhouse gas intensities. *Nat. Sustain.* **3**, 459–462 (2020).

[17] Indian Railways. *Indian Railways Statistical Statements* (Ministry of Statistics and Programme Implementation, Government of India, 2016–2022).

[18] Railway Association of Canada. *Rail Trends* (Railway Association of Canada, 2021).

[19] Machalaba, D. Why railroads are making freight trains longer and longer. *Wall Street J.,* June 15 (2018).

[20] Statistics Bureau. *Japan Statistical Yearbook* (Ministry of Internal Affairs and Communications, Japan, 2022).

[21] *Ministry of Land, Infrastructure and Transport* (Railroad Statistical Yearbook 2020, Republic of Korea, 2021).

[22] Deutsche Bahn. *Facts & Figures 2020* (Deutsche Bahn, 2021).

[23] Office of Rail and Road (ORR). *Table 1310—Freight moved by commodity* https://dataportal.orr.gov.uk/statistics/usage/freight-rail-usage-and-performance/table-1310-freight-moved-by-commodity/ (2022).

[24] Office of Rail and Road (ORR). *Table 1333—Freight train kilometres by operator* https://dataportal.orr.gov.uk/statistics/usage/freight-rail-usage-and-performance/table-1333-freight-train-kilometres-by-operator/ (2022).

[25] Madsen P. M., Dillon R. L., Triantis K. P., Bradley J. A. The relationship between freight train length and the risk of derailment. *Risk Analyses* **44**, 2616–2628 (2024). 10.1111/risa.14312 38807489

[26] Allianz pro Schiene. *740-Meter-Schienennetz: Wie der Güterzug länger werden kann* (Railway Gazette International, 2016).

[27] Railway Gazette International. Longer freight trains to go ahead. 4 October 2017 https://www.railwaygazette.com/freight/longer-freight-trains-to-go-ahead/45230.article (2017).

[28] RailFreight.com. *How Long Until Using Longer Trains?* (RailFreight.com, 2020).

[29] International Union of Railways (UIC). *KORAIL successfully conducts a commercial test of a 50-wagon freight train.* https://uic.org/com/enews/article/korail-successfully-conducts-a-commercial-test-of-a-50-wagon-freight-train (2022).

[30] International Railway Journal (IRJ). *DB Schenker runs 500th 835m-long freight train.* https://www.railjournal.com/freight/db-schenker-runs-500th-835m-long-freight-train/ (2014).

[31] DCVelocity. *AAR, in policy shift, to oppose any effort to change truck size laws.* https://www.dcvelocity.com/articles/29722-aar-in-policy-shift-to-oppose-any-effort-to-change-truck-size-laws (2018).

[32] Federal Highway Administration (FHWA). *Compilation of Existing State Truck Size and Weight Limit Laws – Report to Congress* (US Department of Transportation, 2015).

[33] Lumsden K. Truck Masses and Dimensions – Impact on Transport Efficiency. *ACEA (no date)* https://www.acea.auto/files/SAG_8_Trucks_Masses__Dimensions.pdf.

[34] International Transport Forum (ITF). *Towards Efficient, Safe and Sustainable Road Freight* (International Transport Forum/OECD, 2019).

[35] National Heavy Vehicle Regulator (NHVR). *General Mass and Dimension Limits* (National Heavy Vehicle Regulator, Government of Australia, 2021).

[36] Transportation Research Board (TRB). *Regulation of Weights, Lengths, and Widths of Commercial Motor Vehicles: Special Report 267* (The National Academies Press, 2002).

[37] Liimatainen, H., Pöllänen, M. & Nykänen, L. Impacts of increasing maximum truck weight—case Finland. *Eur. Transp. Res. Rev.* **12**, 1–12 (2020).

[38] Department of the Environment, Transport, and the Regions (DETR). *Efficient JIT Supply Chain Management: Nissan Motor Manufacturing (UK) Ltd*, Good Practice Case Study 374 (1998).

[39] Airbus. *Global Market Forecast 2021-2040* (Airbus, 2021).

[40] Boeing. *Commercial Market Outlook 2021-2040* (Boeing, 2021).

[41] ShipHub. *World’s Biggest Container Ship MSC Irina* (Shiphub, 2024).

[42] Schäfer A. W., Heywood J. B., Jacoby H. D., Waitz I. A. *Transportation in a Climate-Constrained World* (MIT Press, 2009).

[43] Delgado O., Rodríguez F., Muncrief R. *Fuel Efficiency Technology in European Heavy-Duty Vehicles: Baseline and Potential for The 2020–2030 Time Frame* (ICCT, 2017).

[44] Dray, L. et al. Cost and emissions pathways towards net-zero climate impacts in aviation. *Nat. Clim. Change* **12**, 956–962 (2022).

