# Supplementary material for: Energy and economic benefits from economies of scale in intercity freight transportation
Source: NPJ Sustain Mobil Transp. 2025 Mar 28;2(1):13. doi: 10.1038/s44333-025-00028-6 (PMC11953041; doi:10.1038/s44333-025-00028-6)
Supplement: Supplementary file 1 — Supplementary Information [file 44333_2025_28_MOESM1_ESM.pdf]

Supplementary Information: Energy and Economic Benefits from Economies of Scale in Intercity Freight Transportation

Philip Krammer, Andreas W. Schafer

This Supplementary Information describes the database of and regression results from our study. Section 1 describes the underlying data and the cost categories included in transport costs, and Section 2 contains the results of the estimated scale elasticities with respect to unit costs and energy intensity along with the counterfactual exercises.

1. Underlying Data

Our dataset consists of operating statistics from intercity freight carriers at different aggregation levels. The main variables of interest are revenue tonne-km (RTK), available RTK ($RTK_{available}$), vehicle-km travelled (VKT), operating costs and the amount of fuel or energy used.

Supplementary Table 1 provides an overview of the data sources underlying our study. The dataset builds upon 10 countries and two aggregated country groups (EU and the world) over different time periods, altogether encompassing 1925 through 2019; we excluded any later observations due the coronavirus pandemic-induced distortions. All monetary values are converted from local currency units into international US dollars using exchange rates from the World Bank, and subsequently deflated to 2021, using a GDP deflator for the US economy.[1]

Supplementary Table 1: Overview of data sources

**Mode**	**Country**	**Time period**	**Aggre-gation level**	**Data type**	**Pro-prietary data**	**Variable description**			**Incl. in Fig. (F)/Table (T)**				**Reference**	**Worksheet name in Supplementary Data 1 (1) and 2 (2)**
						**Unit Costs**	**Energy Intensity**	**Scale Variable**	**F1**	**F2**	**F3**	**T1,2**		
Air	USA	1991-2019	QTC	Pa	no	OC	F	L,C	yes	yes	yes	yes	2	AirUSA (1)
Air	USA	1949-1980	YC	TS	no	OC		L	no	no	yes	no	3	Air (2)
Road	USA	1999-2003	YTC	CS	no	OC		L[6]	yes	no	no	yes	4	RoadUSA-TL/LTL (1)
Road	USA (a)	1925-1940	YC	TS	no		F	L[6]	no	yes	yes	no	5	RoadUSAa (1)
Road	USA (b)	1989-1998[m]	YC	TS	no		E	L	no	yes	yes	yes	6	RoadUSAb (1)
Road	USA	1941-1988	YC	TS	no			L	no	no	yes	no	7	Road (2)
Road	Australia	1971-2019	YTC	TS	no		E	L	no	yes	yes	yes	8, 9, 10	RoadAUS (1)
Road	Canada	2004-2018	YTC	TS	no	R		L	yes	no	yes	yes	11	RoadCAN (1)
Road	Great Britain	2018	TC	CS	no	OC	F	L	yes	yes	no	yes	12, 13	RoadGBR (1)
Road	Great Britain	1953-2019	YC	TS	no			L	no	no	yes	no	12	Road (2)
Road	EU countries	1997-2009[m]	YC	CS	no		F[2]	L	no	yes	no	yes	14, 15, 16	RoadEU2 (1)
Road	Germany	1952-2019	YC	TS	no			L	no	no	yes	no	17	Road (2)
Road	several	1940-2010[m]	YC	CS	no		E	L	no	yes	no	yes	18	RoadDataGucwaSchafer (1)
Rail	USA	1970-2019	YC	TS	yes	OC	F	L,C	yes	yes	yes	no	19	RailUSA (1)
Rail	USA	1916-2019	YC	TS	no			L	no	no	yes	no	20	Rail (2)
Rail	USA	1990-2019	YTC	Pa	no	OC	F	L,C	yes	yes	yes	yes	21, 22	RailUSA STB (1)
Rail	Canada	1986-2009	YC	TS	no	OC	F	L	yes	yes	yes	no	23	RailCAN (1)
Rail	Canada	2001-2020	YC	TS	no	R	F	L	no	no	yes	no	24	Rail (2)
Rail	India	1951-2019[m]	YC	TS	no	R	F[3]	L	no	no	yes	no	25	Rail (2)
Rail	Germany	1998-2019	YC	TS	no	R	E	L	no	yes	yes	no	26	RailDEU (1)

Supplementary Table 1: Overview of data sources used - continued

Mode	Country	Time period	Aggregation level	Data type	Proprietary data	Variable description			Incl. in Fig. (F)/Table (T)				Reference	Worksheet name in Supplementary Data 1 (1) and 2 (2)
						Unit Costs	Energy Intensity	Scale Variable	F1	F2	F3	T1,2		
Rail	Great Britain	1966-2019[m]	YC	TS	no			L	no	no	yes	no	27, 28, 29	Rail (2)
Rail	Japan	1936-2019[m]	YC	TS	no	R		L	yes	no	yes	no	30	RailJPN (1)
Rail	Russia	1913-2017	YC	TS	no			L	no	no	yes	no	31, 32, 33	Rail (2)
Sea IWW	Netherlands	2004	YC	CS	no		E	L	no	yes	no	no	34	IwwNLD (1)
Sea IWW	France	2009	YC	CS	no		E	L	no	yes	no	no	35	IwwFRA (1)
Sea	World	2010-2012	YT	CS	yes	OC[1]	E[4]	L	yes	yes	no	yes	36, 37, 38, 39	SeaCont (1), SeaBulk (1), SeaOil (1), SeaLPG (1)
Sea	World	2010-2012	YT	CS	no		CO_2	L	no	yes	no	no	38	SeaIMO (1)
Sea	World	1961-2019[m]	YT	TS	no			L	no	no	yes	no	40, 41	Sea (2)
Sea	USA	1960-2019[m]	YT	TS	no			L	no	no	yes	no	42	Sea (2)
Pipe (Oil)	USA	2003-2019	YTC	TS	no	OC	E[5]	L	yes	yes	no	no	43, 44	PipeUSA&CAN (1)
Pipe (Oil)	Canada	1993-2001	YTC	TS	no	OC	E[5]	L	yes	yes	no	no	45	PipeCAN (1)
Pipe (Gas)	USA	2010	YTC	CS	no	OC		L	yes	no	no	no	46	PipeGasUSA (1)

Notes: m = some years missing; aggregation levels: Q = by quarter, Y = by year, T = by vehicle type or carrier type, C = by country; data types: Pa = panel data, CS = cross-sectional data, TS = time series data; Prop. Data = proprietary information/data; variable descriptions: OC = operating costs, R = revenue, F = energy use from fuel use, E = energy use, CO_2 = energy use from CO_2 intensity, L = scale variable RTK/VKT (average load), measuring economies of scale (accounting for economies of fleet size, infrastructure extension, distance, and vehicle size), C = scale variable $RTK_{available}$/VKT (average capacity), measuring economies of scale with respect to vehicle size. 1 = operating costs from Clarkson time charter rates and Moore Stephens operating costs, 2 = energy use from fuel use or CO_2 intensity, 3 = data not extracted, 4 = energy use from IMO fleet efficiency data, 5 = energy use from energy costs and retail price of electricity, 6 = fleet average data, which includes observations of vans and small trucks.

Figure 1 in main text

Air freighter transport. For air freighters, we used air transport data by carrier from the US Bureau of Transportation Statistics.[2] We combined the US Air Carrier Traffic and Capacity Summary by Service Class (T1, monthly data) and Schedules P-6 (quarterly data) and P-12(a) (quarterly data). T1 provides data on RTK and VKT, P-6 on operating costs by category, and P-12(a) on the amount of fuel consumed. We excluded observations with significant changes in a single variable between two consecutive years (e.g., a doubling of the gallons of fuel used while changes in RTK remain small, or sudden changes in transport-related expenses while total operating expenses remain similar). We also excluded observations which violate basic physics (outliers in the U-shaped relationship between the ratio of energy use and available tonne-km plotted over average stage length). The final dataset contains 17 US airfreight carriers from 1990 to 2019. In a final step, we aggregated the quarterly data to yearly data.

Road transport. Our road transport analysis relies on multiple data sources. For the US, we used financial and operating statistics of for-hire trucking companies from the US Federal Motor Carrier Safety Administration, published by the US Bureau of Transportation Statistics.[4] This dataset spans ranges from 1999 to 2003 and contains over 12,000 records of 3,769 different motor carriers. In many cases, however, data is missing or varies widely between two consecutive reporting periods. We excluded observations where non-operating expenses exceed total operating expenses (lack of focus on transport) or where non-operating expenses are negative, observations where transport is partly accomplished by other means (rail, water, and air), observations with the calculated average load larger than 100 tonnes per truck (heavy haul trucking of special freight is therefore not excluded a priori) or smaller than 10 kilograms, and motor carriers with less than 4 observations (to be able to identify outliers from the time series). We also disregard observations with three standard deviations from the mean (observations with a high variation between consecutive reporting periods) and less than 10^{-4} standard deviations from the mean (near-identical observations or repeated data) for average load, operating costs, fuel costs and fuel taxes per RTK. The final dataset contains 1,112 records of 252 motor carriers. The VKT data refers to the total km operated, both loaded and empty. The motor carriers in this data set are classified by general freight (GF), specialty freight (SF), or household goods (HHG) carriers and by their general freight type into truckload (TL), less than truckload (LTL), parcel and container transport carriers. SF carriers are, among others, tank motor carriers (including liquid petroleum and chemical

products) and heavy equipment carriers. Ultimately, we limited the data to TL and LTL carriers above 5 tonnes of average load, which also include GF and SF motor carriers.

In addition, we collected annual average road freight data for Canada (ref [11]) and Great Britain (ref [12]). The Canadian data includes only operating statistics based on revenues (fuel use data is not provided), which we used to approximate total operating costs. The data is split into Local, Domestic, Transborder and Long-Distance Shipments. As we focus on intercity transportation, we excluded the local shipment category from our analysis.

For Great Britain, we collected data from the UK Department for Transport.[12] We combined tables RFS0109, RFS0110, and RFS0125 from ref [12] with published total operating and fuel costs data from IRTE (ref [13]).

Rail transport. The rail dataset consists of aggregate US Class I freight railroads from the Association of American Railroads (ref [19]), individual US Class I freight railroads by railroad company (carrier) from the Surface Transportation Board (refs [21,22]), the two major Canadian freight railroads, Canadian National from 1986 to 2002 and Canadian Pacific from 1986 to 2009 (ref [23]), aggregate Canadian rail freight data from the Railway Association of Canada (ref [24]), and aggregate rail freight data from Japan (ref [30]). For all datasets, we distinguish between train and locomotive-kilometres.

The US Class I freight railroad data (ref [21]) covers the period 1990-2023 and includes seven Class 1 railroads, i.e., Burlington Northern and Santa Fe Railway (BNSF), CSX Transportation, the Grand Trunk Western Railroad (GT), the Kansas City Southern Railway (KCS), the Norfolk Southern Railway (NS), the SOO Line Railroad, and the Union Pacific Railroad (UP).

The Canadian data is based on Table 404-0004 (operating expenses), Table 404-0014 (RTK, locomotive kilometres), and Table 404-0012 (diesel fuel cost) of Statistics Canada.[23] Because Canadian National began to additionally offer passenger services from 2001 (reflected in the aggregate statistics as of 2003), we included data until only 2002. US and Canadian freight railroads are not electrified. The energy intensity for the US and Canada is therefore calculated from the reported amount of fuel used.

Sea transport. Data describing ship operating costs along with the generated RTK is not publicly available. For example, the financial reports of Maersk Line include operating expenditures but not RTK. We therefore relied on time charter (TC) rates by vessel, which

we complemented with average operating statistics from other sources. In particular, we combined TC rates from Clarkson Research Services (ref [36], indicated in equations 1-3 by *, available for 2010-14), with vessel specific data from Clarkson (indicated by **), operating costs data from Moore Stephens LLP (ref [37], indicated by §, 2013 data), fleet performance data (average sea speed, average days at sea) from the Third IMO GHG Study (ref [38], indicated by †, available for 2007-12), and fleet efficiency data (average load factor and average empty running factor) from ref [39] (indicated by ‡, available for 2010-12). The time period common to all observations is 2010-2012. TC rates are date-specific and ship-specific, and we therefore included each ship only once in our data (with a preference for the earliest TC rate observation) to avoid near duplicates originating from the different TC rates per ship. Individual ships are identified by the vessel's name. All variables represent annual average values by vessel type and size category, which we aggregated to average totals by ship-owner.

We calculated RTK from VKT, vehicle size VS, load factor LF, and empty running factor EF using

$$\mathrm{RTK} = \underbrace{\mathrm{speed}^{\dagger} \cdot \mathrm{TC\ days}^{*} \cdot \mathrm{U}^{\dagger}}_{\mathrm{VKT}} \cdot \underbrace{\mathrm{VS}^{*} \cdot (1 - \mathrm{EF}^{\ddagger}) \cdot \mathrm{LF}^{\ddagger}}_{\mathrm{Avg.\ Load}} \tag{1}$$

where VS refers in this case to the ship's deadweight tonnage (DWT) and U is the vehicle's time utilization (derived from the average number of days at sea). Total operating costs are obtained from TC and fuel costs (we used the mean value of the TC period as an approximation for the TC duration in days) using

$$\text{operating costs} = \underbrace{\mathrm{TC\ day\ rate}^{*} \cdot \mathrm{TC\ days}^{*}}_{\mathrm{TC\ cost}} + \text{fuel costs} \tag{2}$$

$$\text{fuel costs} = \mathrm{power}_{\mathrm{ME}}^{**} \cdot \underbrace{\left(\frac{\text{avg. sea speed}^{\dagger}}{\text{design speed}^{**}}\right)^{2}}_{\text{power factor}} \cdot \mathrm{SFOC}_{\mathrm{ME}}^{**} \cdot \mathrm{TC\ days}^{*} \cdot \mathrm{U}^{\dagger} \cdot p_{\mathrm{fuel}}, \tag{3}$$

where p_{fuel} refers to Singapore bunker fuel spot prices of type IFO380 from ref [47] and SFOC to the specific fuel oil consumption of the main engine (ME); the use of auxiliary engines is not considered. SFOC values refer to the operating condition at the vessel's individual design speed. The power factor in the equation accounts for the differences in fuel consumption due to differences in operating speed. In cases where the average sea speed (by ship category) is

greater than the design speed of the ship (as a result of the mean values by ship category), we assumed that average sea speed equals the vessel-specific design speed (power factor = 1).

When transporting liquefied natural gas, liquid ammonia or liquid hydrogen, the storage tanks on liquefied gas tankers produce boil-off gas, which is often used for propulsion (e.g., in dual-fuel-diesel-electric DFDE vessels). This influences the total operating costs (through the fuel costs and boil-off gas costs) as well as the vessel's overall emission intensity. As the boil-off rate and use depends on many different factors, we limited our data sample of liquefied gas tankers to LPG carriers. LPG boil-off has traditionally not been used as a propulsion fuel (ref [48]) and the first vessels with LPG–diesel dual fuel engines commenced operations in 2020 (ref [49]).

Pipeline transport. We collected data related to (crude) oil pipeline operators from the regulatory authorities in the US (ref [43]) for 2000 to 2019 and Canada (ref [44]) for 2000 to 2015. The US gas pipeline data relates to operators for the year 2010.[46] In addition to pipeline data by operator, we obtained aggregate oil pipeline operations data from Statistics Canada (ref [45]) for 1993 to 2001. (Aggregate oil pipeline data for the US also exists (ref [50]), but the number of pump stations is not reported).

We selected oil pipelines by type of product transported. Only trunk line systems, primarily transporting crude oil are included in the dataset. Our FERC/CER oil pipeline dataset consists of the Keystone Pipeline (US, Canada, and combined section), the Trans Mountain Pipeline (Canada section only), the Portland-Montreal pipeline (Canada and US combined), and the Minnesota Pipeline. As a result of modifications to the pipeline infrastructure (number of pumps), electrification and automation measures, as well as the mix of energy carriers (natural gas, petroleum, electricity) used to power the pumps (ref [51]), we excluded the Trans-Alaska Pipeline System. We complemented the FERC and CER data with the number of pump stations from the corresponding company webpages (as cited in the dataset) and with industry electricity prices data from EIA (ref [52]) and Natural Resources Canada (ref [53]).

Pipelines are optimized for a specific flow rate or load factor (defined as the ratio of average load to peak load). To meet the changes in oil demand, the flow rate of the pipeline is adapted, thus either approaching or departing from the optimum design point for minimum energy use. The specific energy use data over the average load for each pipeline therefore indicates a Pareto frontier.[54] Our collected data resembles this trend once the different pipelines are compared against each other. The changes in energy use along the Pareto

frontier affect operating costs in a similar way, but with a reduced impact, given the small share of fuel costs to total costs.

For oil pipelines, the FERC (ref [43]) and CER (ref [44]) data describe operating expenses, transport volumes, energy use (i.e., operating and fuel costs in combination with electricity prices) and the number of pumping stations, which allow calculating unit costs and average load. Unit costs represent total operating costs divided by total tonne-km generated. The average load was calculated by dividing total tonnes by the number of pumping stations, adjusted for the respective pipeline traffic. When pipelines consist of several pipes, the average load is further divided by the number of pipes. For the aggregate Canadian dataset (ref [45]), we divided the average load as calculated for the US by the number of major crude oil pipelines, which we were able to derive from the CER webpage. As this represents a rough approximation, we excluded that dataset from our regressions.

For gas pipelines, we collected data from operators for the year 2010.[46] The dataset consists of 148 gas pipeline operators and contains transmission system miles, the number of compression stations, volumes transported, and economic data, incl. operating costs (energy use is not reported). As data on RTK is not available, we multiplied the calculated average load per compression station with pipeline distance (i.e., the total length of the transmission system) to approximate RTK. We used a minimum and maximum threshold of the calculated distance between compression stations to identify outliers in the data, which we set at 150 and 450 km. Given these data limitations, we did not use the collected pipeline data in our regressions to determine a scale elasticity.

Figure 2 in main text

Air freighter transport. We used RTK, VKT, and energy use data by carrier from Figure 1. In addition, we combined the US Air Carrier Traffic and Capacity Statistics by Aircraft Type (T2, quarterly data) data from BTS (ref [2]) with Schedule P-12(a) data using the aircraft type and the US DOT unique carrier identification number as the matching criteria. As this dataset is available by aircraft type, we selected only freighter aircraft and dedicated airfreight carriers. We excluded observations with non-zero revenue passenger miles and aggregated the data to yearly observations.

Road transport. We collected annual average road freight operating statistics and energy use data from multiple sources for the US (refs [5,6,7]), Australia (refs [8,9,10]), Great Britain (ref [12]; as in Figure 1), and EU countries (refs [14,15,16]). The Australian and Great-Britain data additionally distinguish vehicle category. As we focus on intercity transportation, we excluded the vehicle categories representing small trucks, vans, and light commercial vehicles. (Because the US, Australian, and EU country data include only operating statistics for RTK, VKT, and energy consumption they are excluded in Figure 1).

For three EU countries and the UK (pre-Brexit), we combined published average EU road freight energy intensities from the UK and France (ref [14]), Turkey (ref [15]), and Spain (ref [16]) in a separate dataset. In addition to the above datasets, we included selected published data from ref [18].

Rail transport. The rail dataset consists of aggregate US Class I freight railroads from the Association of American Railroads (ref [19]), the seven US Class I freight railroads (full list see Figure 1) from the Surface Transportation Board (refs [21,22]), the two major Canadian freight railroads, Canadian National from 1986-2002 and Canadian Pacific from 1986-2009 (ref [23]), and the German freight railroad DB Cargo (ref [26]).

DB Cargo directly reports the required key statistics in the DB collection of annual reports (ref [26]), including average vehicle load, RTK, and energy use per tonne-km based on primary energy use.

Inland waterway transport. IWW data by carrier has not been available. We therefore relied on aggregate statistics and included published IWW energy use data from the Netherlands (ref [34]) and France (ref [35]). The data is available by ship category and ship type for the years 2004 and 2009.

Sea transport. We used the data as described in Figure 1. In addition, we included the raw IMO data from ref [38] for comparison (cost data is not available). This data is representative for the global ship fleet and is available by ship type and size category for the years 2010-2012. As in Figure 1 for the TC data, the energy data for container ships indicates the same tendency of higher specific energy intensities at larger average loads given the impact of higher average sea speeds.

Pipeline transport. We used the data as in Figure 1 but without the natural gas pipeline data due to missing energy use information.[46] To convert oil pipeline electricity use into energy

use, we used a primary energy factor of 2.62 kWh/MJ for the year 2019. We derived it from US EIA (ref [55]), by dividing the primary consumption for electric power (minus imports) by the electricity net generation for that year.

Figure 3 in main text

Air freighter transport. We used BTS (ref [2]) T1 data by carrier to obtain a time series of average load of the entire cargo fleet from 1974 to 2021. We complemented this time series with 1949-1980 data from ref [3].

Road transport. For Germany, we extracted RTK and VKT data from "Verkehr in Zahlen" (ref [17]), to calculate a time series of average load. For Canada, we included data from Statistics Canada (ref [11]) as in Figure 1. For Great Britain, we combined the tables TSGB0401 and TRA0201 (TSGB0702) from DfT (ref [12]). For Australia, we included data for articulated trucks as in Figure 1. For the US, we combined the time series of average load from refs [5,6,7].

Rail transport. The rail dataset consists of aggregate US Class I freight railroad statistics from 1916 to 2019 (ref [20]), aggregate Canadian freight railroad data (refs [23,24]), summary statistics of Indian Railways (ref [25]), aggregate statistics of the German freight railroad DB Cargo (ref [26]), UK rail freight transport statistics data (refs [27,28,29]), and data from Japan (ref [30]) and Russia (refs [31,32,33]).

Sea transport. We collected time series data of the fleet average DWT by ship category from the UNCTAD Review of Maritime Transport reports (ref [40]) and the UNCTAD statistics database (ref [41]). Additionally, we included the fleet average DWT of the US Flag Merchant Fleet from BTS (ref [42]). Given that fleet utilization is not available in aggregated UNCTAD and BTS data, all sea data in Figure 3 is shown as fleet average DWT per ship (instead of fleet average load).

Table 1 in main text

Same data sources as in Figures 1 and 2, starting in 1990.

Table 2 in main text

Air freighter transport. We used air transport data by carrier from the US Bureau of Transportation Statistics (ref [2]) as described and shown in Figures 1 and 2, to estimate both the scale elasticity of unit costs per tonne-km and the scale elasticity of energy intensity.

Road transport. For the scale elasticity of unit costs per tonne-km (Supplementary Table 3), we used Canadian intercity domestic and long-distance transport between 2004 and 2018 from Statistics Canada (ref [11]), US intercity TL transport between 1999 and 2003 from BTS (ref [4]), both as described and shown in Figure 1. For the scale elasticity of energy intensity (Supplementary Table 4), we used data from the US (ref [6], for the years 1989-1990), Great Britain (ref [12], as in Figure 1, for the year 2018), four European countries (refs [14,15,16], including Great Britain, France, Turkey, Spain, with observations between 1997 and 2007), and road data from ref [18] (2013; including France, Great Britain, Switzerland, and the US, with observations between 1990 and 2010), as described and shown in Figure 2. In addition, we used Australian articulated and rigid trucks data between 1985 and 2019 from BITRE (refs [8,9]) and ABS (ref [10]) as described and shown in Figure 2, to estimate the scale elasticity of energy intensity for Australian trucks.

Rail transport. We used the seven individual US Class I railroad companies from the Surface Transportation Board (refs [21,22]) as described and shown in Figures 1 and 2, to estimate both the scale elasticity of unit costs per tonne-km and the scale elasticity of energy intensity.

Sea transport. We used the data as described and shown in Figures 1 and 2, to estimate both the scale elasticity of unit costs per tonne-km and the scale elasticity of energy intensity. As the final LPG dataset contains only 12 observations, we excluded the LPG carriers' group from our regressions.

Supplementary Table 2 includes a detailed description of the cost categories included in transport costs. While intercity freight transportation may also be multi-modal, our analysis merely considers single-mode transportation. This restriction is imposed by the data available, which builds upon total operating expenditures of freight transport companies.

Supplementary Table 2: Overview of cost elements included in the data set

**Type of freight and data source**	**Variable costs**	**Fixed costs**
Air (USA), data as in Figure 1	aircraft fuel and oil, food, traffic commissions, landing fees, rentals, transport related expenses	salaries and benefits, maintenance material, advertising, communication, insurance, outside flight equipment maintenance, depreciation, amortization
Road (USA), TL[1] data as in Figure 1 from ref [4]	operating supplies (fuel, oil, outside maintenance, vehicle parts, tires and tubes), fuel taxes, operating taxes and licenses, equipment rentals, purchased transportation	salaries and benefits, insurance, depreciation, amortization, communications and utilities, building and office equipment rents,
Rail (CAN), data as in Figure 1	rail operation (administration, fuel expenses, train control expenses, equipment cleaning and servicing, casualties and claims), rolling-stock maintenance (locomotives, cars, intermodal/work equipment), rentals, taxes	infrastructure maintenance (tracks, buildings, signals, communications, power, terminals, fuel stations), infrastructure amortization (tracks, buildings, signals, communications, power, terminals, fuel stations), rolling-stock amortization (locomotives, cars, intermodal and work equipment), salaries and benefits
Sea (material costs unassigned and excluding port charges), data as in Figure 1	fuel costs	labour costs, capital costs
Pipeline (USA), data as in Figure 1 for US crude oil pipelines	operating fuel and power, rentals, taxes	salaries and wages, outside services, depreciation and amortization, general expenses not classified as variable costs

Notes: 1) Here, we only use the TL data as it provides the dataset with the largest trucks and largest average loads, to better indicate the differences in fixed and variable costs in comparison to the other transport modes.

2. Scale Elasticities

Supplementary Tables 3 and 4 contain the regression results of the scale elasticities with respect to unit costs and energy intensity using the model specification outlined in the Methods section. Each row represents a separate regression.

We then used the estimated coefficients to determine the contribution of EoS to the overall reductions of energy intensity using counterfactuals (a similar analysis for unit costs was not possible due to data limitations). Supplementary Table 5 contains the results.

Supplementary Table 3: Regression results for cost economies of scale

No.	Transport mode	Time period	ln avg. load (tonnes)	ln avg. capacity (tonnes)	ln avg. load factor	ln avg. speed (kts)	ln avg. fuel price ($/unit[9])	Year	Fixed effects	Const.	R^2	N
1	Air (USA)	1991-2019	-0.91(-40.2)				0.27(7.95)	d	no	2.31(30.8)	0.68	749
2	Air (USA)	1991-2019	-1.07(-12.1)				0.29(8.15)	d	yes[1]	1.89(9.34)	0.74	749
3	Air (USA)	1991-2019		-0.92(-33.8)	-0.84(-11.2)		0.28(7.98)	d	no	2.40(19.5)	0.70	749
4	Air (USA)	1991-2019		-1.36(-10.6)	-0.98(-10.6)		0.29(8.29)	d[7]	yes[1]	2.48(8.95)	0.74	749
5	Road (CAN)	2004-2018	-0.63(-7.41)				0.58(14.4)	d	yes[3]	-3.1(-10.0)	0.94	30
6	Road (CAN)	2004-2018	-0.67(-8.10)		-0.38(-2.03)[2]		0.55(13.4)	d	yes[3]	-3.0(-10.6)	0.95	30
7	Road (USA[4])	1999-2003	-0.88(-13.6)					d	yes[5]	-0.02(-0.1)	0.97	158
8	Rail (USA)	1990-2019	-0.95(-17.1)				0.14(6.65)	d[7]	yes[5]	2.38(6.28)	0.83	205
9	Rail (USA)	1990-2019		-0.98(-17.4)[6]	-1.38(-7.13)[2]		0.13(5.87)	d[7]	yes[5]	2.37(6.34)	0.83	205
10	Sea Bulk	2010-2012	-0.75(-77.2)			0.85(2.25)	d[7]	FE[8]	no	-0.08(-0.1)	0.88	872
11	Sea Container	2010-2012	-0.57(-39.9)			0.95(10.7)	0.64(16.6)	FE[8]	no	-6.7(-23.1)	0.78	1,355
12	Sea Oil	2011-2012	-0.75(-57.4)			d[7]	d[7]	FE[8]	no	2.15(15.2)	0.95	179

Notes: Estimated models using ln transport cost in $/tonne-km as the dependent variable. Each line represents a separate regression using data selected from the data sources as described in the section for Table 1 in the Supplementary Information. t-statistics are in parenthesis. Summary statistics provided in Table 1 in the main text. d: dropped. [1] by aircraft type, consisting of 31 different aircraft subgroups. [2] using average utilization derived from empty running. [3] by transport category, including domestic and long-distance shipments. [4] above 5 tonnes average load. [5] by transport company. [6] using capacity derived from ‘1 - empty running’. [7] variables dropped as estimated coefficients have an incorrect sign. [8] using year fixed effects instead of a continuous variable given the short time period. [9] Air (USA): US$/gallon, road (CAN): CA$/liter, rail (USA): US$/gallon, sea: US$/tonne.

Supplementary Table 4: Regression results for energy economies of scale

No.	Transport mode	Time period	ln avg. load (tonnes)	ln avg. capacity (tonnes)	ln avg. load factor	ln avg. speed (kts)	Year	Fixed effects	Const.	R^2	N
13	Air (USA)	1991-2019	-0.59(-50.8)				-0.007(-6.30)	no	18.2(8.44)	0.78	771
14	Air (USA)	1991-2019	-0.77(-22.5)				d	yes[1]	4.47(56.2)	0.89	771
15	Air (USA)	1991-2019		-0.53(-37.8)	-0.85(-22.0)		-0.009(-8.44)	no	23.0(10.4)	0.79	771
16	Air (USA)	1991-2019		-0.76(-15.2)	-0.77(-21.3)		d	yes[1]	4.46(40.7)	0.89	771
17	Road (six countries[2])	1990-2018	-0.49 (-19.8)				d	yes[3]	1.21(17.8)	0.88	124
18	Road (AUS[4])	1985-2019	-0.61(-171)				-0.002(-6.56)	no	5.70(9.83)	0.99	70
19	Rail (USA)	1990-2019	-0.77(-21.2)				-0.003(-3.48)	yes[8]	9.49(6.57)	0.88	205
20	Rail (USA)	1990-2019		-0.77(-20.3)[5]	-0.73(-6.86)[6]		-0.003(-3.47)	yes[8]	9.48(6.55)	0.88	205
21	Sea Bulk	2010-2012	-0.55(-49.5)			d[7]	FE[9]	no	3.16(27.4)	0.75	872
22	Sea Container	2010-2012	-0.40(-27.2)			1.22(13.4)	FE[9]	no	-1.34(-9.6)	0.49	1,355
23	Sea Oil	2011-2012	-0.58(-30.4)			1.63(3.20)	FE[9]	no	-0.45(-0.39)	0.90	179

Notes: Estimated models using ln energy intensity in MJ/tonne-km as the dependent variable. Each line represents a separate regression using data selected from the data sources as described in the section for Table 1 in the Supplementary Information. t-statistics are in parenthesis. Summary statistics provided in Table 1 in the main text. d: dropped. [1] by aircraft type, consisting of 31 different aircraft subgroups. [2] US, Great Britain, France, Turkey, Spain, and Switzerland. [3] by country / data source. [4] articulated and rigid trucks. [5] using capacity derived from '1 - empty running'. [6] using average utilization derived from empty running. [7] variable dropped as estimated coefficients have an incorrect sign. [8] by transport company. [9] using year fixed effects instead of a continuous variable given the short time period.

Supplementary Table 5: Changes in average load and energy intensity

Transport mode	Equation number[1]	Time period	Changes in average load		Changes in energy intensity		
			total % change over time period	% change p.a. over time period	Total % change over time period	% change p.a. over time period	% change of total as a result of EoS
Air (USA)	13	1991-2019	60%	1.7%	-38%	-1.7%	65%
Air (USA, cap. & LF[2])	15	1991-2019	60%	1.7%	-38%	-1.7%	50%
Air (USA, cap. only[3])	15	1991-2019	60%	1.7%	-38%	-1.7%	72%
Road (AUS)	18	1985-2019	46%	1.1%	-27%	-0.9%	80%
Rail (USA)	19	1990-2019	62%	1.7%	-37%	-1.6%	85%
Rail (USA, cap. & LF[2])	20	1990-2019	62%	1.7%	-37%	-1.6%	85%
Rail (USA, cap. only[3])	20	1990-2019	62%	1.7%	-37%	-1.6%	86%

Notes: [1] reference to first column in Supplementary Table 4. [2] considering both changes in capacity and load factor. [3] only considering changes in capacity.

Supplementary References

1. World Bank. *Open Data*. https://data.worldbank.org/ (2022).
2. Bureau of Transportation Statistics (BTS). *Air Carrier Summary Data (Form 41)* http://www.transtats.bts.gov (1991-2019).
3. Air Transport Association of America (ATA). *Facts & Figures about Air Transportation* https://www.airlines.org/who-we-are/history/ (1957-1981).
4. Bureau of Transportation Statistics (BTS). *Motor Carrier Financial and Operating Information*. Motor Carrier Operators: Annual Report http://www.transtats.bts.gov (1999-2003).
5. Barger H. The transportation industries, 1889-1946: a study of output, employment, and productivity (NBER Books, 1951).
6. Davis S.C. Transportation energy data book: Editions 12-20. Office of Transportation Technologies, US Department of Energy (1992-2000).
7. American Trucking Association (ATA). American Trucking Trends, several editions (1941-1988).
8. Bureau of Infrastructure and Transport Research Economics (BITRE). Australian Government, *Report 88 Greenhouse Gas Emissions from Australian Transport: Long Term Projections* https://www.bitre.gov.au/sites/default/files/report_088.pdf (1995).
9. Bureau of Infrastructure and Transport Research Economics (BITRE). Australian Government, *Motor vehicles, Australia, January 2022* (first issue) https://www.bitre.gov.au/publications/2022/motor-vehicles-australia-january-2022-first-issue (2022).
10. Australian Bureau of Statistics (ABS). Survey of Motor Vehicle Use (several years) (1998-2018).
11. Statistics Canada. *Table 23-10-0219-01. Trucking commodity industry activities* https://www150.statcan.gc.ca/t1/tbl1/en/tv.action?pid=2310021901 (2020).
12. UK Department for Transport (DfT). *Statistics at DfT* https://www.gov.uk/government/organisations/department-for-transport/about/statistics (2023).
13. Institute of Road Transport Engineers (IRTE). Transport Engineer, January 2019, pp.22-23 (2019).

14. Léonardi J., Rizet C., Browne M., Allen J., Pérez-Martínez P.J., Worth R.W. Improving energy efficiency in the road freight transport sector: the application of a vehicle approach (Liverpool University Press, 2008).
15. Ozen M., Tuydes-Yaman H. Evaluation of emission cost of inefficiency in road freight transportation in Turkey. Energy Policy **62**, 625-636 (2013).
16. Pérez-Martínez P.J. The vehicle approach for freight road transport energy and environmental analysis in Spain. European Transport Research Review **1**(2), 75-85 (2009).
17. Bundesministerium für Verkehr und digitale Infrastruktur (BMDV). *Verkehr in Zahlen* https://bmdv.bund.de/SharedDocs/DE/Artikel/G/verkehr-in-zahlen.html (1952-2023).
18. Gucwa M., Schäfer A. The impact of scale on energy intensity in freight transportation. Transportation Research Part D: Transport and Environment **23**, 41-49 (2013).
19. Association of American Railroads (AAR). Railroad Ten-Year Trends, Washington, DC (1987-2021).
20. Association of American Railroads (AAR). Railroad Facts. Washington, DC (1934-2021).
21. Surface Transportation Board (STB). *Economic Data, Annual Report Financial Data*, several ed. https://www.stb.gov/reports-data/economic-data/annual-report-financial-data/ (1990-2023).
22. US National Archives. US National Archives and Records Administration, *Carrier Annual Reports* https://catalog.archives.gov/search-within/30010072?q=UP (2024).
23. Statistics Canada. *Table 404-0004* (operating expenses), *Table 404-0014* (RTK, locomotive kilometers), and *Table 404-0012* (diesel fuel cost) https://www.statcan.gc.ca/ (2019).
24. Railway Association of Canada (RAC). *Rail Trends* https://www.railcan.ca/ (2011-2021).
25. Indian Railways. *Indian Railways Statistical Statements* http://www.indianrailways.gov.in (2016-2022).
26. Deutsche Bahn. Annual reports consisting of: *Integrated Report, Facts & Figures Report, Sustainability Report* https://ir.deutschebahn.com/ (2007-2019).
27. UK Department for Transport. *Transport Statistics Great Britain*, TSGB 1996 & 1986 statistical dataset https://www.gov.uk/government/statistical-data-sets/ (1986, 1996).

28. Network Rail. *Annual Reports* https://www.networkrail.co.uk/who-we-are/publications-and-resources/regulatory-and-licensing/annual-return/ (1999-2022).
29. Office of Rail and Road. *National Statistics* https://www.data.gov.uk/ (2020).
30. Japan Statistical Yearbook. Ministry of Internal Affairs and Communications, Statistics Bureau, *Rail Freight Transport, incl. Japan Railways (JR)*, several ed. https://www.stat.go.jp/ (2011-2023).
31. Macheret D.A., Kudryavtseva A.V. Retrospective Analysis of Efficiency of Railway Freight Operations. World of Transport and Transportation **16**(4), 102–115 (2018).
32. US Department of Commerce (DOC). *USA/USSR: Facts and Figures 1991*, Economic and Statistics Administration, Bureau of the Census (1991).
33. USSR Transportation and Communication. Statistical Compilation, State Statistical Publishing House, Moscow (1957, 1972).
34. Schilperoord H.A. *Environmental performance of inland shipping*. Report prepared for Royal HaskoningDHV, Centraal Bureau Rijn- en Binnenvaart Koninklijke Schuttevaer. Project Nr. 9P1060 http://www.ebu-uenf.org/fileupload/rapport_milieuperformance.pdf (2004).
35. Ministère De L'Écologie, Du Développement Durable, Des Transports Et Du Logement (MEEDDAT). Etude De L'Efficacite Energetique Et Environnementale Du Transporte Maritime, MEEDDAT, Paris (2009).
36. Clarkson Research Services (Clarkson). *Shipping intelligence network* https://sin.clarksons.net/ (2014).
37. Moore Stephens. *OpCost*. Moore Stephens LLP https://opcost.moorestephens.org/ (2015).
38. Smith T.W.P., Jalkanen J.P., Anderson B.A., et. al. *Third IMO GHG Study 2014*. International Maritime Organization (IMO), London, UK https://docs.imo.org (2015).
39. Smith T.W.P., Prakash V., Aldous L., Krammer P. *The Existing Shipping Fleet's CO2 Efficiency*. International Maritime Organization (IMO), London, UK https://docs.imo.org (2015).
40. UNCTAD. Review of Maritime Transport, UNCTAD, several ed. (1968-2019).
41. UNCTAD. *UNCTAD Statistics* https://unctadstat.unctad.org/ (2023).
42. Bureau of Transportation Statistics (BTS). *Number and Size of the U.S. Flag Merchant Fleet and Its Share of the World Fleet* https://www.bts.gov/content/number-and-size-us-flag-merchant-fleet-and-its-share-world-fleet (2024).

43. Federal Energy Regulatory Commission (FERC). *Form No. 6 - Annual Report of Oil Pipeline Companies* https://www.ferc.gov/industries-data/oil/general-information/oil-industry-forms (2020).
44. Canada Energy Regulator (CER). *Pipeline Profiles* https://www.cer-rec.gc.ca/en/data-analysis/energy-commodities/pipeline-profiles/index.html (2020).
45. Statistics Canada. *Pipeline Transportation of Crude Oil and Refined Petroleum Products*, Tables: 55-201-X https://www150.statcan.gc.ca/n1/en/catalogue/55-201-X (2020).
46. Smith C.E. Natural gas pipeline operators' 2010 profits reach record levels. Oil & gas journal, **109**(16), 92-92 (2011).
47. Bunkerworld. *Bunker fuel spot prices* http://www.bunkerworld.com/ (2015).
48. International Chamber of Shipping (ICS). Tanker Safety Guide: Liquefied Gas, 3rd ed. Marisec. (2018).
49. S&P. *BW Gemini first of 12 VLGCs to retrofit with dual-fuel propulsion in China shipyard*: BW LPG. S&P Global Platts, 7. Oct. 2020 https://www.spglobal.com/platts/ (2020).
50. Bureau of Transportation Statistics (BTS). *Oil Pipeline Profile* https://www.bts.gov/content/oil-pipeline-profile (2020).
51. Trans Alaska Pipeline System (TAPS). *Facts*. Alyeska Pipeline Service Company https://www.alyeska-pipe.com/assets/uploads/pagestructure/TAPS_PipelineFacts/editor_uploads/2016FactBook.pdf (2016).
52. US Energy Information Administration (EIA). *Electricity* https://www.eia.gov/electricity/data/browser/ (2020).
53. Natural Resources Canada. *Industrial Energy Prices and Background Indicator*, Government of Canada https://oee.rncan.gc.ca/corporate/statistics/neud/dpa/showTable.cfm?type=HB&sector=agg&juris=00&rn=7&page=6 (2020).
54. Hooker J.N. Oil pipeline energy consumption and efficiency (No. ORNL-5697). Oak Ridge National Lab., TN (USA) (1981).
55. US Energy Information Administration (EIA). *Total Energy* https://www.eia.gov/totalenergy/data/browser/ (2020).
